# Supplementary material for: Patterns of Intron Gain and Loss in Fungi
Source: PLoS Biol. 2004 Nov 30;2(12):e422. doi: 10.1371/journal.pbio.0020422 (PMC532390; doi:10.1371/journal.pbio.0020422)
Supplement: Table S1 — Also available at http://genes.mit.edu/NielsenEtAl/. (4.3 MB ZIP). [file pbio.0020422.st001.zip › NielsenEtAl/html/236.html]

AN4409.1.NCU01667.1.MG01102.1.FG06281.1


```
 CLUSTAL W (1.82) Multiple Sequence Alignments - Introns Inserted


Sequence 1: MG01102.1	351 aa
Sequence 2: FG06281.1	336 aa
Sequence 3: NCU01667.1	363 aa
Sequence 4: AN4409.1	397 aa
Alignment Length: 403 aa
Number Identitical Residues: 196 aa
Alignment Score (without introns) 8894


MG01102.1 	------------------------------------MRPSTLR-AINRALAGNEARTYS-
NCU01667.1	-----------------------------------MMSRATTR-TIKSAVGQIQARSVSN
FG06281.1 	------------------------------------MKTSTFR-IARGAAAGLQKRAYTK
AN4409.1  	MSHSRIISQLFVDALVLSTISFRPKSFKFFNIYILALPMASLRSVLKSQSLRHTVRSYSS
          	 : :   :.   .:   :: :  ..: .  .     :  :: *:  .        *: :.

MG01102.1 	------SSASPRHLMSIADLTPTELTTLVRSAATHKHAVKSGAGA-----PLHLAQSLTG
NCU01667.1	S----AASSTPRHLLSISQLSPAEFSKLVLNASAYKQATKAAFAAGPGQVPRTLDGKLKG
FG06281.1 	------ATTAPRHLMTIADLTPTEFANLVRDANTRKIAVK--------------------
AN4409.1  	QTMPPASPFAPRHFLSIADLSPSEFATLVRNASSHKRAIKSGSMP------QNLQGSLLG
          	.: ..::. :***:::*::*:*:*::.** .* : * * *:.  .           .  .

MG01102.1 	KTVA~MMFSKRSTRTRVSTEAAVAMMGGHPMFLGKDDIQLG0VNESLLDTSTVISSMTSC
NCU01667.1	RTVA~MMFSKRSTRTRVSTEAAVALMGGHPMFLGKDDIQLG0VNESLYDTSKVISSMTSC
FG06281.1 	---A1MMFSKRSTRTRVSTEAAVTMLGGHPMFLGKDDIQLG0VNESLYDTSKVISSMTSC
AN4409.1  	KTVA~MIFSKRSTRTRVSTEGAVVQMGGHPMFLGKDDIQLG~VNESLYDTSVVISSMVSC
          	 : * *:*************.**. :*************** ***** *** *****.**

MG01102.1 	MVARVGPHSDVTGLAKHSSVPVINALSDDFHPLQTIADFLTIHSHHPSSTPG--SLGLEG
NCU01667.1	MVARVGPHSDVADLARDSSVPVINALSDDFHPLQAIADFQTIHEAFATKPAHEASLGLEG
FG06281.1 	MVARVGPHSDVATLAKHSSVPVINALSDDFHPLQTIADFLTLAETFPASTSKGATLGLNG
AN4409.1  	IVARVGKHAEVADLAKHSSVPVINALCDSFHPLQAVADFQTIYEAFTPKAHHLSSLGLEG
          	:***** *::*: **:.*********.*.*****::*** *: . .....   ::***:*

MG01102.1 	LKIAWIGDSNNVLFDLALGAAKLGCHVAVASPTGYGIPENMRTLIQSASKASGSGGSLSE
NCU01667.1	MKVAWVGDSNNVLFDMATACVMLGVDIAVASPAGYGIPDRMKQIILSVAEGVAKPGKLIE
FG06281.1 	LKVAWVGDANNVLFDLAIGCVKMGVDISVAAPKGYEIPDAMRQLITSAGDGLSSPGKLIE
AN4409.1  	LKIAWVGDANNVLFDMAIAATKMGVDIAVATPKGYEIPPHMLELIKSAGEGVSKPGKLLQ
          	:*:**:**:******:* ... :* .::**:* ** **  *  :* *.... .. *.* :

MG01102.1 	TTVPEEAVKDADILVTDTWVSMGQEAEAKKRLAAFAGFQITNDLAKRGGAKKDWKFMHCL
NCU01667.1	TTVPEEAVKDADILVTDTWVSMGQEAETQRRLKAFAGFQITNELAKRGGAKPGWKFMHCL
FG06281.1 	TNVPEEAVKDANVLVTDTWVSMGQEADTQKRLKDFAGFQITNDLAKRGGAKADWKFMHCL
AN4409.1  	TNIPEEAVKDADILVTDTWVSMGQEEEKAQRLKEFDGFQITAELAKRGGAKEGWKFMHCL
          	*.:********::************ :  :**  * ***** :******** .*******

MG01102.1 	PRHPEEVHDEVFYSPRSLVFEEAENRLWAAVA1ALEAFVVNKGKI-
NCU01667.1	PRHPEEVDDEVFYGPQSLVFPEAENRLWAAVS1ALEAFVVNNGRIL
FG06281.1 	PRHPEEVADEVFYSPRSLVFPEAENRLWAALS1ALEGFVVNKGKF-
AN4409.1  	PRHPEEVSDEVFYSNRSLVFPEAENRLWAAIS~ALEGFVVNKGKIE
          	******* *****. :**** *********:: ***.****:*::
```
